# Deep learning-based reconstruction for three-dimensional volumetric brain MRI: a qualitative and quantitative assessment

**DOI:** 10.1186/s12880-025-01647-8

**Published:** 2025-03-27

**Authors:** Yeseul Kang, Sang-Young Kim, Jun Hwee Kim, Nak-Hoon Son, Chae Jung Park

**Affiliations:** 1 Department of Radiology, Yongin Severance Hospital, Yonsei University College of Medicine, 363 Dongbaekjukjeon-daero, Giheung-gu, Yongin-si, Gyeonggi-do 16995 Republic of Korea; 2 MR Clinical Science, Philips Healthcare, Seoul, Republic of Korea; 3 Department of Statistics, Keimyung University, Daegu, Republic of Korea

**Keywords:** Magnetic resonance imaging, Deep learning, Compressed sensing, Qualitative assessment, Quantitative assessment

## Abstract

**Background:**

To evaluate the performance of a deep learning reconstruction (DLR) based on Adaptive-Compressed sensing (CS)-Network for brain MRI and validate it in a clinical setting.

**Methods:**

Ten healthy volunteers and 22 consecutive patients were prospectively enrolled. Volunteers underwent 3D brain MRI including T1 without CS factor (9:16 min, reference standard); with CS factor of 2 without DLR (CS2, 4:6 min); with CS factor of 2 with DLR (DLR-CS2); with CS factor of 4 without DLR (CS4, 2:6 min); and with CS factor of 4 with DLR (DLR-CS4). The patients’ MRI included the CS2 and DLR-CS4. The volumes of lateral ventricles, hippocampus, choroid plexus, and white matter hypointensity were calculated and compared among the sequences. Three radiologists independently assessed anatomical conspicuity, overall image quality, artifacts, signal-to-noise ratio (SNR), and sharpness using a 5-point scale for each sequence.

**Results:**

Applying acceleration factors of 2 and 4 reduced the scan time to 65.4% and 33.5%, respectively, of that of the reference standard. Volumes of all the measured subregions showed no significant differences among different sequences in all participants. In qualitative analysis, the interrater agreement was excellent (κ = 0.844–0.926). In volunteers, quality of DLR-CS4 were comparable to those of CS2 for all metrics except for the overall image quality and SNR despite a 51.2% scan time reduction. In patients, DLR-CS4 showed quality comparable to that of CS2 for all metrics.

**Conclusions:**

DLR allowed the scan time reduction by at least half without sacrificing image quality and volumetric quantification accuracy, supporting its reliability and efficiency.

**Supplementary Information:**

The online version contains supplementary material available at 10.1186/s12880-025-01647-8.

## Introduction

Three-dimensional (3D) high-resolution structural MRI is fundamental to evaluate various neurological diseases. 3D T1-weighted imaging is a basic MRI sequence used for the evaluation of structural anatomy and volumetric analysis. To obtain acceptable image quality in a limited scan time, many accelerating techniques have been developed, including parallel imaging, compressed sensing, and partial Fourier [1, 2]. However, these techniques with high acceleration factors frequently and inevitably introduce noise and degrade spatial resolution.

Recently, deep learning (DL) has been suggested for the reconstruction of accelerated MR scans as well as for image denoising, gaining increased attention in recent years [3–6]. MRI scanners equipped with DL reconstruction (DLR) for denoising are already available for clinical use. One of the recently developed DLR methods called Adaptive-Compressed sensing (CS)-Network utilizes a novel convolutional neural networks (CNN) to integrate and enhance conventional CS-SENSE algorithms (i.e., CS + SENSE) [7]. As the traditional CS-based reconstruction results in compromised diagnostic value due to difficulties in choosing the optimal sparsity transforms [8], DL has replaced wavelet-based sparsity transform with multi-scale sparsifying transform in this novel method. With this strategy, the performance of denoising can be improved through automatically optimized the sparsity constraint during the iterative reconstruction.

Recent clinical studies using either the prototype or commercial versions of the DLR based on Adaptive-CS-Network have demonstrated improved image quality in the imaging studies of the prostate [9], musculoskeletal area [10, 11], breasts [12], heart [13], and MR cholangiopancreatography [14] and proved its feasibility; however, it has not been validated yet in the field of neuroradiology. We, therefore, aimed to evaluate the performance of a novel DLR tool in adult brain 3D T1-weighted images (T1) with quantitative and qualitative assessments and to validate its feasibility and clinical usefulness in a prospective setting.

## Materials and methods

### Participants

The Institutional Review Board of our tertiary institution approved this prospective study (No. 9-2024-0091). Informed consents were obtained from all participants. This study adhered both the Declaration of Helsinki and the Health Insurance Portability and Accountability Act (HIPAA). In this prospective study, 10 healthy volunteers (median age: 38 [interquartile range, IQR, 31–39] years; male: female, 4:6) and 22 consecutive patients (median age: 76 [IQR 74–79] years; male: female, 9:13) undergoing clinical brain MRI at our tertiary institution were recruited. The patients were imaged for a variety of clinical indications: subjective cognitive decline (*n* = 12), mild cognitive impairment (*n* = 1), Alzheimer’s disease (*n* = 2), Parkinson’s disease (*n* = 1), infarction (*n* = 2), and other conditions, including visual disturbance, trauma, carotid artery stenosis, and hydrocephalus.

### MRI acquisition

All MRI examinations were performed using a 3T MR scanner (Ingenia Elition; Philips Healthcare, Amsterdam, Netherlands) equipped with 32-channel receiver head coil. Different imaging protocols were used for healthy volunteers and patients. Detailed MRI parameters are presented in Supplementary Material. For the healthy volunteers, five different 3D T1-weighted images (T1) were obtained as follows: (1) 3D T1 without CS factor (reference standard, 9 min 16 s); (2) 3D T1 with CS factor of 2 without DLR (CS2, 4 min 6 s); (3) 3D T1 with CS factor of 2 with DLR (DLR-CS2, 4 min 6 s); (4) 3D T1 with CS factor of 4 without DLR (CS4, 2 min 6 s); and (5) 3D T1 with CS factor of 4 with DLR (DLR-CS4, 2 min 6 s). Twenty-two patients underwent brain MRI, including imaging with two 3D T1 sequences, as follows: (1) CS2 (4 min 6 s) and (2) DLR-CS4 (2 min 6 s). CS2 is a standard brain MRI sequence used at our institution. Therefore, patients additionally underwent a faster 3D T1 scan by increasing the CS factor to 4, which was then reconstructed with DLR (DLR-CS4) to evaluate the feasibility of DLR in the patient population.

### DLR

The DLR used in this study builds on parallel imaging SENSE combined with CS, CS-SENSE in which the prior information such as coil sensitivity profile and low-resolution background information is used for data reliability during the iterative reconstruction [15]. The applied DL, Adaptive-CS-Network was first introduced for the FastMRI challenge to reconstruct under-sampled knee MRI data [7] and it is now extended to all image contrasts and application domains. Briefly, the Adaptive-CS-Net is an advancement of the deep learning–based iterative shrinkage-thresholding algorithm (ISTA) network proposed by Zhang and Ghanem [16]. The power of Adaptive-CS-Net comes from the use of multi-scale and multi-slice computations in iterative learning-based reconstruction scheme in which MR images are efficiently denoised by multiple reconstruction blocks. Similar to CS-SENSE reconstruction, the network is fed by raw k-space data, coil sensitivity and coarse background information, but the sparsity constraining step of the iterative reconstruction is DL-based. In each individual block of the network, data consistency checking with the incoming raw k-space data is performed. The Adaptive-CS-Net used in this study was initially trained with a large dataset from various anatomical regions and acceleration factors. The algorithm was refined to run on standard reconstruction hardware, in contrast with a previously reported network [7].

### Qualitative analysis

To qualitatively compare image quality among different sequences, two neuroradiologists (C.J.P. and J.H.K., with 4 and 5 years of experience in neuroradiology, respectively) and one junior resident evaluated the following four image quality parameters on a 5-point Likert scale (1 = unacceptable; 2 = poor; 3 = acceptable; 4 = good; 5 = excellent): anatomical conspicuity, overall image quality, artifacts, signal-to-noise ratio (SNR), and sharpness. The anatomic conspicuity was assessed by evaluating the delineation of existing pathologies contrast to the adjacent normal brain structures or delineation of subregions of deep gray matter in cases with no abnormalities on 3D T1. Gray-white matter differentiation was used to evaluate sharpness. The reviewers evaluated all image series in the Picture Archiving and Communication Systems database. No restrictions were applied to window-level setting adjustments regarding the time or ability to scroll through the images. The reviewers were blinded to patient information, including disease and scan parameters, to identify the type of sequence.

### Quantitative analysis

For quantitative analysis, we obtained the volumes of lateral ventricle (LV), hippocampus, choroid plexus (CP), white matter hypointensity (WMH), amygdala, accumbens, and total intracranial volume (ICV) from each sequence using FastSurfer software. The reasons we chose these structures for analysis are as follows: (1) LV and hippocampus are frequently evaluated in studying neurodegenerative diseases, where the extent of volume loss or atrophy of specific brain regions matter [17–19]; (2) WMH is known to have a significant detrimental effect on cognitive decline and dementia, therefore, we include WMH in our analysis since accurate measurement of WMH volume matters; (3) CP has recently gathered attention and increasingly investigated due to possible association between CP volume and clinical status in patients with Alzheimer’s disease and Parkinson’s disease [17, 20, 21]; (4) The volumes of amygdala and accumbens, which are relatively small structures with small volumes, were taken into consideration to evaluate the effect of DLR on small structures. FastSurfer is a fast and extensively validated DL pipeline for fully automated processing of structural human brain MRIs (Henschel et al., NeuroImage 2020; 219:117012). This enabled whole-brain segmentation into 95 classes within 50 s per participant. The details and validity thereof can be found elsewhere (https://deep-mi.org/research/fastsurfer/*).* The regional volume was expressed as the ratio of the regional volume to the total ICV (ratio to the total ICV × 10^3^).

### Statistical analysis

Based on the results of the Shapiro–Wilk test, a comparative test was conducted using parametric and non-parametric methods. Qualitative analysis results were compared between the different sequences using the Kruskal–Wallis test and Mann–Whitney U test for healthy volunteers and patients, respectively. The interrater agreement between the three radiologists was evaluated on a total of 94 images from all study participants (*n* = 32) using the weighted kappa (κ). A κ value ≤ 0.20 indicated slight agreement; 0.21–0.40, fair agreement; 0.41–0.60, moderate agreement; 0.61–0.80, substantial agreement; and 0.81–0.99, almost perfect agreement [22]. Quantitative volumetric results were compared using an analysis of variance and independent two-sample t-tests for healthy volunteers and patients, respectively. A Bland–Altman analysis was performed to assess the quantitative volumetric biomarker equivalence of the datasets. Statistical analyses were performed using SAS software (version 9.4, SAS Institute). Results with a two-tailed P value < 0.05 were considered statistically significant. The Dwass–Steel–Critchlow–Fligner correction method was used to prevent an increase in type 1 errors in multiple testing using the post hoc method for the Kruskal–Wallis test.

## Results

By applying CS factors of 2 and 4 to the reference standard, the scan time was reduced to 65.4% and 33.5%, respectively. Comparing CS2 and CS4, CS4 enables faster scanning, with a scan time which is 51.2% of that of CS2.

### Qualitative results

**Fig. 1:**
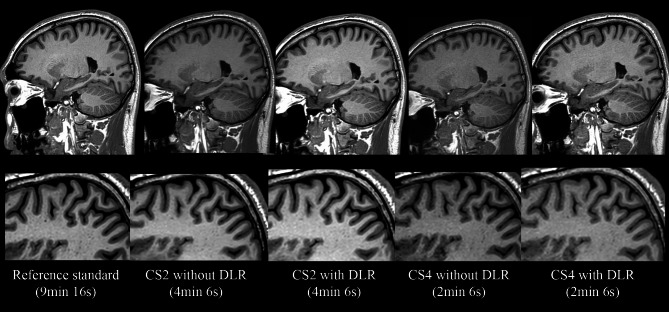
Representative figures from a healthy volunteer (upper row: coverage of the whole brain; lower low: magnification of cortical/subcortical areas). Due to denoising of deep learning reconstruction (DLR), the signal-to-noise ratio significantly increased in CS2 with DLR (DLR-CS2) compared with CS2 without DLR and in CS4 with DLR (DLR-CS4) compared with CS4 without DLR. DLR-CS2 provided images with quality comparable to that of the reference standard. CS4 without DLR showed the worst results for qualitative analysis among the five sequences, with notable artifacts in the white matter and reduced sharpness in the border between the gray and white matter. Applying DLR to CS4 (DLR-CS4) enhanced the image quality by decreasing artifacts in the white matter and increasing sharpness in the border between gray and white matter, making its quality comparable to that of CS2. It is notable that DLR-CS4 and CS2 achieved similar qualitative analysis results, whereas the scan time of DLR-CS4 was reduced by approximately half (2 min 6 s vs. 4 min 6 s)

The weighted κ values that assessed interrater agreement showed almost perfect agreement in anatomic conspicuity, overall image quality, artifacts, SNR, and sharpness (ranging from 0.844 to 0.926, Table 1). In healthy volunteers, applying a CS factor of 2 to the reference standard significantly degraded the quality of all metrics (P-values < 0.05) except SNR (P value = 0.241). When DLR was applied to CS2, it significantly improved the quality of all metrics to the extent of the reference standard (median score of 5 for all metrics, [IQR, 4–5], Table 2). CS4 exhibited the worst performance among the five sequences, with median scores of 3 for all metrics, which were significantly lower than those of CS2 (all P-values < 0.001, Table 2). CS4 images frequently showed blurriness between the cortical gray matter, subcortical white matter, and grainy artifacts in the white matter (Fig. 1). When DLR was applied to CS4 (DLR-CS4), the quality of all metrics was significantly improved, compared with CS4 (all P-values < 0.001), with decreased artifacts (median 4, [IQR 4–5]), improved anatomic conspicuity (median 4, [IQR 4–4]), overall image quality (median 4, [IQR 4–4]), sharpness (median 4, [IQR 4–4]), and increased SNR (median 4.5 [IQR 4–5]). In addition, when comparing DLR-CS4 with CS2, DLR-CS4 achieved comparable quality in anatomic conspicuity (DLR-CS4 vs. CS2, median 4 [IQR, 4–4] vs. median 4 [IQR, 4–4]), artifacts (median 4 [IQR, 4–5] vs. median 5 [IQR, 4–5]), and sharpness (median 4 [IQR, 4–4] vs. median 4 [IQR, 4–4]). However, the overall image quality was significantly better with CS2 (median 5 [IQR 5–5]) than with DLR-CS4 (median 4 IQR [4–4]). SNR was also significantly higher in CS2 (median 5 [IQR 5–5]) than DLR-CS4 (median 4.5 IQR [4–5]). (*P* = 0.021, Table 2; Fig. 1). The detailed P values from comparisons of qualitative analysis results among sequences are presented in the Supplementary Table S1.

**Table 1:** Interrater agreement

Total 94 images from both volunteers + patients (*n* = 32)	Rater A	Rater B	Kappa	95% confidence interval	*P* value
Anatomic conspicuity	Rater 1	Rater 2	0.888	(0.806, 0.971)	< 0.001
	Rater 1	Rater 3	0.904	(0.828, 0.981)	< 0.001
	Rater 2	Rater 3	0.920	(0.850, 0.999)	< 0.001
Overall image quality	Rater 1	Rater 2	0.926	(0.863, 0.989)	< 0.001
	Rater 1	Rater 3	0.924	(0.858, 0.990)	< 0.001
	Rater 2	Rater 3	0.880	(0.801 0.959)	< 0.001
Artifacts	Rater 1	Rater 2	0.923	(0.856, 0.990)	< 0.001
	Rater 1	Rater 3	0.865	(0.779, 0.951)	< 0.001
	Rater 2	Rater 3	0.910	(0.840, 0.980)	< 0.001
Signal-to-noise ratio	Rater 1	Rater 2	0.922	(0.845, 0.995)	< 0.0001
	Rater 1	Rater 3	0.883	(0.794, 0.973)	< 0.0001
	Rater 2	Rater 3	0.851	(0.752, 0.951)	< 0.0001
Sharpness	Rater 1	Rater 2	0.890	(0.811, 0.969)	< 0.001
	Rater 1	Rater 3	0.844	(0.752, 0.936)	< 0.001
	Rater 2	Rater 3	0.892	(0.813, 0.971)	< 0.001

**Table 2:** Multirater quantitative assessments in volunteers

Rating criterion	Reference standard		CS2		DLR-CS2		CS4		DLR-CS4		*P* value	
	Median	IQR	Median	IQR	Median	IQR	Median	IQR	Median	IQR	CS2 vs. CS4	CS2 vs. DLR-CS4
Anatomic conspicuity	5	(5,5)	4	(4,4)	5	(5,5)	3	(3,3)	4	(4,4)	< 0.001	0.241
Overall image quality	5	(5,5)	5	(5,5)	5	(5,5)	3	(3,3)	4	(4,4)	< 0.001	< 0.001
Artifacts	5	(5,5)	5	(4,5)	5	(5,5)	3	(3,3)	4	(4,5)	< 0.001	0.123
Signal-to-noise ratio	5	(5,5)	5	(5,5)	5	(5,5)	3	(3,4)	4.5	(4,5)	< 0.001	0.021
Sharpness	5	(5,5)	4	(4,4)	5	(5,5)	3	(3,3)	4	(4,4)	< 0.001	0.887

CS2, compressed sensing factor 2 without deep learning reconstruction; DLR-CS2, compressed sensing factor 2 with deep learning reconstruction; CS4, compressed sensing factor 4 without deep learning reconstruction; DLR-CS4, compressed sensing factor 4 with deep learning reconstruction; IQR, interquartile range

**Fig. 2:**
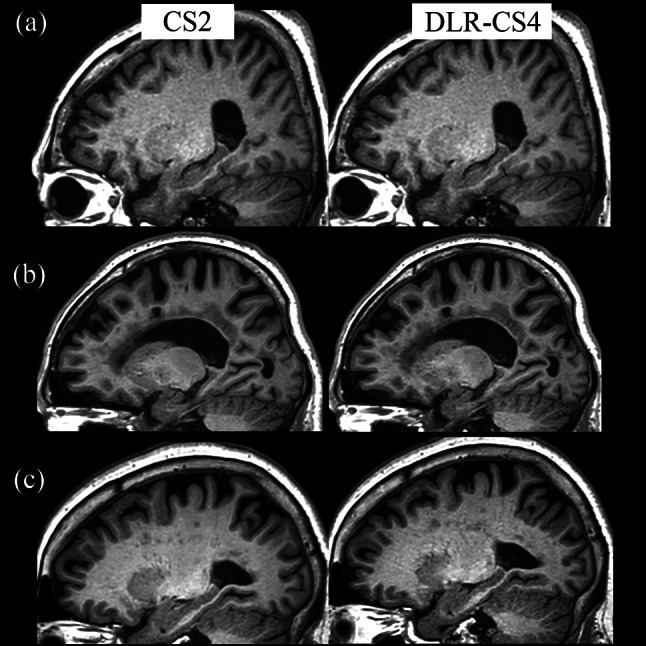
Representative figures from three patients (left: 3D T1 with CS factor of 2 without DLR [CS2]; right: 3D T1 with CS factor of 4 with DLR [DLR-CS4]). (**a**) Patient 1: All the metrics were comparable as per the qualitative analysis results, including artifacts diffusely scattered in the white matters and gray-white matter differentiation. (**b**) Patient 2: The delineation of old infarctions in the corona radiata did not differ significantly between the two sequences. (**c**) Patient 3: Diffuse, grainy artifacts throughout the white matter were significantly worse in DLR-CS4 compared with CS2; meanwhile the other metrics did not differ significantly

In the patient group, DLR-CS4 achieved comparable quality for all metrics, compared with CS2, despite a scan time reduction by approximately half, with all median scores ranging from 4 to 5 (Table 3; Fig. 2). Anatomic conspicuity between pathologies, such as old infarctions or ischemic lesions and normal adjacent white matter was compensated for in DLR-CS4 by applying DLR, which yielded comparable scores of anatomic conspicuity between CS2 and DLR-CS4. Diffuse and grainy artifacts, which were frequently observed in CS4, were also decreased by applying DLR to CS4 at a level similar to that of CS2. SNR decrease which was observed in CS4 was also increased by applying DLR (Fig. 2).

**Table 3:** Multirater quantitative assessments in patients

Rating criterion	CS2		DLR-CS4		*P* value
	Median	IQR	Median	IQR	
Anatomic conspicuity	4.5	(4, 5)	4	(4, 5)	0.864
Overall image quality	4	(4, 5)	4	(4, 5)	0.188
Artifacts	4	(4, 5)	4	(4, 4)	0.226
Signal-to-noise ratio	5	(4, 5)	5	(4, 5)	0.320
Sharpness	4	(4, 5)	4	(4, 5)	0.057

CS2, compressed sensing factor 2 without deep learning reconstruction; DLR-CS4, compressed sensing factor 4 with deep learning reconstruction; IQR, interquartile range

### Quantitative results

**Fig. 3:**
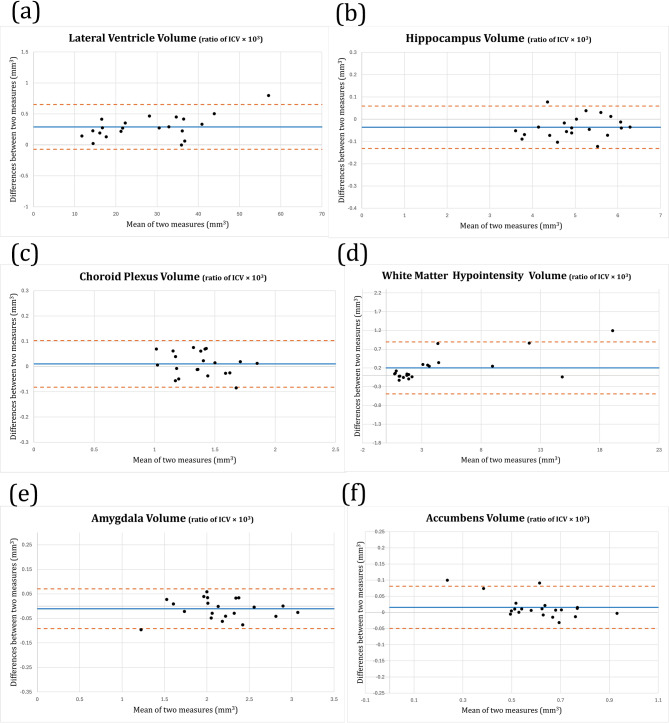
Bland–Altman results for CS2 versus DLR-CS4. The plots demonstrate a linear distribution without significant scatter, indicating consistent concordance between CS2 and DLR-CS4 in the quantitative assessment of volumes of lateral ventricle, hippocampus, choroid plexus, white matter hypointensity, amygdala and accumbens

The volumes of LV, hippocampus, CP, WMH, amygdala, and accumbens were not significantly different among the different 3D T1 sequences in both healthy volunteers and patients (P values = 0.657–0.999) (Table 4). The Bland–Altman plot graph analysis further demonstrated a strong agreement between the quantitative values from CS2 and DLR-CS4 (Fig. 3).

**Table 4:** Quantitative assessments in both volunteers and patients

Regional volumes	Reference standard		CS2		DLR-CS2		CS4		DLR-CS4		*P* value
	Mean	SD	Mean	SD	Mean	SD	Mean	SD	Mean	SD	
**Volunteers**											
LV volume, ratio of ICV × 10^3^	10.913	4.340	11.035	4.206	10.939	4.294	10.866	4.280	10.938	4.280	> 0.999^*^
Hippocampal volume, ratio of ICV × 10^3^	5.880	0.927	6.032	0.907	5.877	0.900	5.903	0.912	5.867	0.909	0.999^*^
CP volume, ratio of ICV × 10^3^	1.007	0.227	1.020	0.221	1.004	0.231	1.002	0.232	1.005	0.237	0.999^*^
WMH volume, ratio of ICV × 10^3^	0.587	0.136	0.622	0.128	0.607	0.140	0.635	0.156	0.614	0.153	0.960^*^
Amygdala volume, ratio of ICV × 10^3^	2.555	0.429	2.675	0.479	2.600	0.447	2.583	0.450	2.581	0.441	0.928^*^
Accumbens volume, ratio of ICV × 10^3^	0.805	0.102	0.833	0.107	0.812	0.106	0.799	0.110	0.798	0.114	0.657^*^
**Patients**											
LV volume, ratio of ICV × 10^3^			27.740	11.921					28.030	12.023	0.938^†^
Hippocampal volume, ratio of ICV × 10^3^			5.006	0.795					4.970	0.804	0.943^†^
CP volume, ratio of ICV × 10^3^			1.385	0.221					1.375	0.232	0.884^†^
WMH volume, ratio of ICV × 10^3^			4.206	4.977					4.407	5.217	0.899^†^
Amygdala volume, ratio of ICV × 10^3^			2.172	0.450					2.162	0.450	0.938^†^
Accumbens volume, ratio of ICV × 10^3^			0.600	0.160					0.616	0.141	0.738^†^

LV, lateral ventricle; CP, choroid plexus; WMH, white matter hypointensity; ICV, intracranial volume; CS2, compressed sensing factor 2 without deep learning reconstruction; DLR-CS2, compressed sensing factor 2 with deep learning reconstruction; CS4, compressed sensing factor 4 without deep learning reconstruction; DLR-CS4, compressed sensing factor 4 with deep learning reconstruction; SD, standard deviation

Calculated from ^*^ANOVA and ^†^independent two sample t-test

## Discussion

We investigated whether compressed sensing-based DLR is a feasible tool for brain imaging by applying different acceleration factors with and without DLR and comparing different sequences for both healthy volunteers and patients. The major findings were as follows: (1) DL-reconstructed CS2 images achieved comparable and excellent quality for all metrics compared with the reference standard, with an approximately 60% reduced scan time; (2) DL-reconstructed CS4 images achieved a quality comparable to CS2 images in approximately half the scan time in both healthy volunteers and patients; and (3) volumetric analysis results did not differ significantly when either different accelerations or DLR were applied. These findings suggest that DLR enables acceptable quality in a significantly reduced scan time and provides accurate volumetric analysis results, which makes it a feasible tool for integration into clinical settings for brain imaging.

Recently, various commercially available DLR tools have been developed and have attracted interest because they produce high-quality images with improved SNR in reduced scan times [23]. Studies have investigated the reliability of the DLR tools for general clinical use in various imaging fields, and several DL-based reconstructions applied to brain MRI showed promising results, by improving the image quality or aiding diagnosis in brain imaging. The vendor-agnostic DICOM-based DL-enhancement software has proven its value because it provides acceptable image quality for 3D brain MRI sequences with a significant scan time reduction in both healthy controls and patients [24, 25]. One of the vendor-driven DLRs which uses CNN to reconstruct the image directly from *k-*space data has been applied to the sella MRI in the postoperative setting after pituitary adenoma surgery and compared with a 3-mm sequence, which revealed that the 1-mm sequence with DLR showed higher diagnostic performance in identifying cavernous sinus invasion and comparable diagnostic performance in identifying residual tumors [5]. This software has also been validated for use in pediatric brain MRI; it can reduce noise and truncation artifacts, and improve lesion conspicuity and overall image quality in T2-weighted images [26]. Another vendor-driven DLR tool based on Adaptive-CS-Network been recently developed and its clinical utility has been proved in recent clinical MRI studies of the head and neck [27], prostate [9], musculoskeletal area [10, 11], breasts [12], heart [13], and MR cholangiopancreatography [14]; however, little is known about its usefulness in the field of brain imaging. Because the validation of Adaptive-CS-Network DLR is still a prerequisite for its clinical use in brain imaging, we applied it to 3D T1 images in brain MRI, with different acceleration factors. For healthy volunteers, 3D T1 images with different CS factors (0, reference standard; 2, and 4) were obtained to evaluate the extent to which DLR can improve the image quality in an accelerated scan, with different CS factors. For patients, an accelerated 3D T1 with a CS factor of 4 was additionally obtained for comparison with a standard 3D T1 with a CS factor of 2. We found that, despite a significant scan time reduction by increasing the CS factor, image quality was maintained by applying DLR. Furthermore, the accuracy of the volumetric analysis results using 3D T1 was maintained when DLR was applied. Considering the high clinical demands for brain MRI acquisition in a hectic schedule, DLR can be suggested as a feasible and efficient tool for integration into brain MRI to reduce the scan time without sacrificing image quality.

Because acquiring 3D T1 images without the CS factor (reference standard) is not practical due to its long scan time (9 min 16 s), CS2 was the standard 3D T1 sequence in our institution. It is clinically important to determine whether we can further reduce the scan time by approximately half by increasing the CS factor to 4 while simultaneously maintaining image quality by applying DLR. We observed that the qualitative analysis results of images acquired using CS2 and DLR-CS4 were comparable between healthy volunteers and patients. For healthy volunteer scans, the overall image quality and SNR of CS2 was significantly better than that of DLR-CS4 and all other quality metrics were comparable between the two sequences. Considering that all the quality metrics of CS4 were significantly worse than those of CS2, DLR had a significant impact on the improvement in the qualitative analysis results. The values of denoising and improved image quality were validated in our patients’ scans, and all quality metrics showed no significant differences between CS2 and DLR-CS4. Therefore, DLR can be safely applied to the standard 3D T1 sequence while reducing the scan time by approximately half, by increasing the CS factor.

The DLR tool used in this study achieved improved image quality through iterative image processing using a DL-based image-sparsifying approach for denoising and artifact [7]. In our study, we observed that the artifacts were substantially reduced by applying DLR. DLR-CS4 frequently showed diffuse, grainy artifacts across the images and throughout the white matter, resulting in a median score of 3 in the qualitative assessment. In general, the introduction of noise and artifacts is inevitable when scan time is reduced by increasing the CS factor. These artifacts decreased when DLR was applied, thereby increasing the score to a level similar to that of CS2. For patient scans, artifacts were present in both 3D T1 with CS2 and DLR-CS4; however, they did not hinder brain pathology or interrupt the reading of MRIs. Therefore, we suggest that DLR can successfully remove the artifacts. Several reports suggest that DLR denoising itself can cause specific artifacts or may enhance artifacts due to the high signal-to-noise ratio of DLR [23]. However, we did not notice any peculiar artifacts caused by or related to DLR in our study. Further studies focusing on the evaluation of the prominent artifacts caused by DLR denoising should be performed.

Quantitative volumetric MR analytical tools are widely used to evaluate patients with variable clinical diagnoses, including neurodegenerative diseases. Accurate results are important for clinical and research purposes. In the present study, we utilized FastSurfer, a fast pipeline for the neuroanatomical surfaces, which has been reported to outperform FreeSurfer with respect to runtime, reliability, and sensitivity [28]. This approach provides a full FreeSurfer alternative for volumetric analysis in a markedly reduced time, while maintaining segmentation accuracy and test-retest reliability, making it more feasible than FreeSurfer [28, 29]. Therefore, it is necessary to validate whether DL-enhanced 3D T1 images provide accurate volumetric analysis results using FastSurfer, as DLR will become more widely used in the future. In our study, we observed no significant differences in the volumes of the brain subregions obtained from different sequences (different acceleration factors with and without DLR). In particular, we chose the hippocampus, WMH, and CP for volumetric analysis together with the lateral ventricles and ICV, as these are frequently investigated in various neurological diseases, including neurodegenerative diseases [20, 30, 31]. Furthermore, amygdala and accumbens with relatively small volumes were also included in our analysis. A previous study reported that there were significant SNR drops in these structures when applying CS, probably due to their small sizes, resulting in volume inconsistencies among different scans [32]. Herein, we compared the volumes of amygdala and accumbens to evaluate whether DLR significantly affects volumetric analysis particularly in the small structures. As a result, we observed that there were no significant differences among different scans, therefore, it can be concluded that DLR does not affect the accuracy of volumetric analysis results from 3D T1-weighted images. The volumes calculated from DL-enhanced T1 images can be safely used for both clinical and research purposes.

This study has some limitations. First, this was a prospective single-center study with a relatively small number of patients. Our results need to be validated in future studies with larger sample sizes and multiple institutions. Second, we only focused on 3D high-resolution T1-weighted images, which are the fundamental sequences for the evaluation of brain structural anatomy, midline structures, and volumetric analysis. Because 3D T2-weighted and fluid-attenuated inversion recovery images are essential for the evaluation of brain pathology, the application of DLR to these sequences should be evaluated in the future. Third, we did not calculate the values of SNR or contrast-to-noise ratio for the specific lesion in our study. Instead, we assessed image SNR and sharpness by radiologists in the qualitative analysis, and observed DLR increased SNR and maintained sharpness. Fourth, whether applying CS-based DLR in a same scan time can improve diagnostic performance in patients with a specific disease was not evaluated in this study. Rather, we focused on the efficiency that DLR might provide, whether it can maintain image quality in a significant reduced scan time, therefore, can be a good solution for tight MRI schedules. Fifth, we only utilized one software (FastSurfer) for volumetric analysis. Since there are many different software for volumetric analysis, future studies using other volumetric analysis software need to be performed to evaluate generalizability of DLR. Lastly, in future studies, this CS-based DLR can be applied to patients with a specific disease and can be validated whether it can improve diagnostic performance or help neuroradiologists’ decisions.

In conclusion, the novel DLR tool based on Adaptive-CS-Network in combination with an accelerated MRI protocol allows significant scan time reduction while maintaining image quality and high volumetric quantification accuracy compared with the standard sequence. Our study supports the reliability and efficiency of DL-based reconstruction, which can be safely incorporated into clinical practice.

## Electronic supplementary material

Below is the link to the electronic supplementary material.

Supplementary Material 1

## Data Availability

The datasets used and/or analysed during the current study are available from the corresponding author on reasonable request.

## Abbreviations

CNN: Convolutional neural network
CP: Choroid plexus
CS: Compressed sensing
DL: Deep learning
DLR: Deep learning reconstruction
ICV: Intracranial volume
ISTA: Iterative shrinkage-thresholding algorithm
LV: Lateral ventricle
WMH: White matter hypointensity
