# Supplementary material for: Deep learning-based reconstruction for three-dimensional volumetric brain MRI: a qualitative and quantitative assessment
Source: BMC Med Imaging. 2025 Mar 27;25:102. doi: 10.1186/s12880-025-01647-8 (PMC11951731; doi:10.1186/s12880-025-01647-8)
Supplement: Supplementary file 1 — Supplementary Material 1 [file 12880_2025_1647_MOESM1_ESM.docx]

**Supplementary Materials**

**Supplementary Material S1. MRI acquisition**

MRI data were acquired using a 3T MRI scanner (Ingenia Elition X or Ingenia CX, Philips Healthcare, Best, the Netherlands) with 32-channel head coil. Different imaging protocols were set for the healthy volunteers and patients, respectively.

For the healthy volunteers, five different 3D T1-weighted images (T1) (TR = 4.5 msec; TE = 2.0 msec; slice thickness = 1.0 mm; flip angle = 8º; acquisition matrix = 240×240; FOV = 200×200 mm^2^) were obtained as follows: 1) 3D T1 without compressed sensing (CS) factor (reference standard, 9min 16s); 2) 3D T1 with CS factor of 2 *without* DLR (CS2, 4min 6s); 3) 3D T1 with CS factor of 2 *with* DLR (DLR-CS2, 4min 6s); 4) 3D T1 with CS factor of 4 *without* DLR (CS4, 2min 6s); and 5) 3D T1 with CS factor of 4 *with* DLR (DLR-CS4, 2min 6s).

For the patients, the protocols were as follows: sagittal 3D T1 with axial and coronal reconstruction, sagittal 3D fluid-attenuated inversion recovery (FLAIR) image with axial reconstruction, 3D axial T2-weighted image (T2) and susceptibility-weighted imaging (SWI). The imaging parameters were as follows: 1) 3D T1: TR = 4.5 msec; TE = 2.0 msec; slice thickness = 1.0 mm; flip angle = 8º; acquisition matrix = 240×240; FOV = 200×200 mm^2^; 2) 3D FLAIR: TR = 4800 msec; TE = 297 msec; slice thickness = 1.0 mm; flip angle = 90º; FOV = 200×200 mm^2^; 3) 3D T2: TR = 2500 msec; TE = 240 msec; slice thickness = 2 mm; flip angle = 90º; FOV = 250×250 mm^2^; 4) SWI: multi-echo fast-field-echo sequence, TR = 51 msec; total 6 echoes; first TE = 0 msec; echo interval = 6.0 msec; slice thickness = 2 mm; flip angle = 20º; FOV = 230×230 mm^2^. For 3D T1, two different 3D T1 sequences were obtained as follows: 1) CS2 (4min 6s); and 2) DLR-CS4 (2min 6s). CS2 is one of our routine sequences in brain MRI in our institution. Therefore, the patients additionally performed a faster 3D T1 scan by increasing CS factor to 4, which was then reconstructed with DLR (DLR-CS4) for the evaluation of feasibility of DLR in the patient population.

**Supplementary Table. P values from comparisons of qualitative analysis results among five different sequences in volunteers**

| Variable |  | CS2 | DLR-CS2 | CS4 | DLR-CS4 |
| --- | --- | --- | --- | --- | --- |
| Anatomic conspicuity | Reference standard | <.001 | 0.241 | <.001 | <.001 |
|  | CS2 |  | <.001 | <.001 | 0.241 |
|  | DLR-CS2 |  |  | <.001 | <.001 |
|  | CS4 |  |  |  | <.001 |
| Overall image quality | Reference standard | 0.042 | 0.396 | <.001 | <.001 |
|  | CS2 |  | 0.644 | <.001 | <.001 |
|  | DLR-CS2 |  |  | <.001 | <.001 |
|  | CS4 |  |  |  | <.001 |
| Artifacts | Reference standard | <.001 | 0.396 | <.001 | <.001 |
|  | CS2 |  | 0.189 | <.001 | 0.123 |
|  | DLR-CS2 |  |  | <.001 | <.001 |
|  | CS4 |  |  |  | <.001 |
| Sharpness | Reference standard | <.001 | 0.241 | <.001 | <.001 |
|  | CS2 |  | <.001 | <.001 | 0.887 |
|  | DLR-CS2 |  |  | <.001 | <.001 |
|  | CS4 |  |  |  | <.001 |

CS2, compressed sensing factor of 2 without deep learning reconstruction; DLR-CS2, compressed sensing factor of 2 with deep learning reconstruction; CS4, compressed sensing factor of 4 without deep learning reconstruction
